# Blue Light Signaling Regulates Escherichia coli W1688 Biofilm Formation and l-Threonine Production

**DOI:** 10.1128/spectrum.02460-22

**Published:** 2022-09-27

**Authors:** Wenjun Sun, Shuqi Shi, Jiao Chen, Wei Zhao, Tianpeng Chen, Guoxiong Li, Kaijie Zhang, Bin Yu, Dong Liu, Yong Chen, Hanjie Ying, Pingkai Ouyang

**Affiliations:** a National Engineering Research Center for Biotechnology, College of Biotechnology and Pharmaceutical Engineering, Nanjing Tech University, Nanjing, China; b State Key Laboratory of Materials-Oriented Chemical Engineering, College of Biotechnology and Pharmaceutical Engineering, Nanjing Tech University, Nanjing, China; c School of Chemical Engineering and Energy, Zhengzhou University, Zhengzhou, China; University of Guelph

**Keywords:** biofilm, *Escherichia coli*, blue light signaling, optogenetics, l-threonine

## Abstract

Escherichia coli biofilm may form naturally on biotic and abiotic surfaces; this represents a promising approach for efficient biochemical production in industrial fermentation. Recently, industrial exploitation of the advantages of optogenetics, such as simple operation, high spatiotemporal control, and programmability, for regulation of biofilm formation has garnered considerable attention. In this study, we used the blue light signaling-induced optogenetic system Magnet in an E. coli biofilm-based immobilized fermentation system to produce l-threonine in sufficient quantity. Blue light signaling significantly affected the phenotype of E. coli W1688. A series of biofilm-related experiments confirmed the inhibitory effect of blue light signaling on E. coli W1688 biofilm. Subsequently, a strain lacking a blue light-sensing protein (YcgF) was constructed via genetic engineering, which substantially reduced the inhibitory effect of blue light signaling on biofilm. A high-efficiency biofilm-forming system, Magnet, was constructed, which enhanced bacterial aggregation and biofilm formation. Furthermore, l-threonine production was increased from 10.12 to 16.57 g/L during immobilized fermentation, and the fermentation period was shortened by 6 h.

**IMPORTANCE** We confirmed the mechanism underlying the inhibitory effects of blue light signaling on E. coli biofilm formation and constructed a strain lacking a blue light-sensing protein; this mitigated the aforementioned effects of blue light signaling and ensured normal fermentation performance. Furthermore, this study elucidated that the blue light signaling-induced optogenetic system Magnet effectively regulates E. coli biofilm formation and contributes to l-threonine production. This study not only enriches the mechanism of blue light signaling to regulate E. coli biofilm formation but also provides a theoretical basis and feasibility reference for the application of optogenetics technology in biofilm-based immobilized fermentation systems.

## INTRODUCTION

Biofilm is a special community comprising microbial cells and their extracellular matrix formed on biotic and abiotic surfaces; it has importance in various fields, such as the industrial and medical sectors (1, 2). Biofilms are widely used to increase the productivity and stability of a process in the biotechnology industry (3). Compared with free-cell fermentation, immobilized fermentation limits cell movement in a fixed space, eliminates the expensive process of cell recovery, reduces production costs, achieves high volumetric productivity, and effectively improves fermentation performance (3–5). Escherichia coli, Saccharomyces cerevisiae, Aspergillus niger, and Corynebacterium glutamicum are specifically used for immobilized continuous fermentation to produce various biochemicals (6–11).

l-Threonine not only is one of the eight essential amino acids in the human body but also is one of the three major amino acids produced during fermentation (12). It has been widely used in food, pharmaceutical, agriculture, cosmetics, and other industries (13). Our previous study has demonstrated that the immobilized fermentation of E. coli W1688 is accompanied by biofilm formation, which effectively improves l-threonine yield (14).

Optogenetics entails the use of light to perform various actions, such as controlling gene expression, regulating protein-protein interactions, and starting systems; optogenetics is associated with advantages such as simple and convenient operation, high spatiotemporal control, programmability, and tunability (14–17). The exploitation of optogenetics to regulate E. coli biofilm formation is a promising approach for enhancing immobilized fermentation. Recent evidence shows the use of optogenetics as a robust tool to study and regulate the key aspects of bacterial biology in a fast and often reversible manner (18). However, the influence of light on various features of bacterial cells remains underexplored (19). Currently, several studies are being conducted focusing on the application of various optogenetic tools in different model organisms (20, 21). In the 1970s, Francis Crick et al. proposed the application of light-controlled systems in eukaryotic systems. With the advancement of optogenetic technology, several optogenetic systems have emerged. For instance, the Magnet system is widely used to modulate various cellular functions, such as protein localization and activity, receptor activation, gene expression, and cell adhesion through photoswitchable protein interactions, which comprise two distinct vivid protein variants—one positively charged (pMag) and one negatively charged (nMag). The two proteins bind via electrostatic interactions, resulting in selective and reversible light-induced heterodimerization and manipulating cellular functions, such as protein-protein interactions and genome editing, through the Magnet system (22–26). In E. coli, expression of the blue light-responsive protein nMag-pMag regulates bacterial adhesion and biofilm formation (23, 24, 27). Therefore, the use of optogenetics (the Magnet system) in biofilm-based immobilized fermentation appears to be feasible.

The phenomenon that blue light affects the growth phenotype of E. coli was identified via the Magnet system used to regulate E. coli biofilm formation; this system was previously neglected in optogenetics. Mark Gomelsky et al. reported that bacteria can sense light and light signaling alters their lifestyle (28). In addition, Natalia Tschowri et al. reported that E. coli senses blue light signaling through the BluF-EAL protein BluF (YcgF) (29). Under the action of blue light signaling, formation of the sugar surface components colanic acid and curli fibers is regulated by the Ymg/Rcs pathway (29–31); the levels of cyclic di-GMP (c-di-GMP) and the biofilm regulator CsgD may also be controlled to regulate the formation of curli fibers in E. coli (31).

In this study, we analyzed the molecular mechanism underlying the inhibitory effects of blue light signaling on E. coli biofilm formation. The findings of this study may provide a theoretical basis for the construction of a strain in which the negative effects of blue light signaling on biofilm formation are alleviated; this strain can further use its blue light-induced genetic tools. For this, we used the Magnet system to enhance biofilm formation in an immobilized fermentation system to achieve blue light-reversible control of the biofilm adhesion and desorption in the early stage of its formation and to realize the directional regulation of blue light signaling on the immobilized fermentation of E. coli. Combined with the natural properties of biofilm and its key role in industrial immobilized fermentation, the problem of slow biofilm formation in immobilized continuous fermentation systems is solved. The findings of this study may expand our understanding of the mechanism of blue light signaling regulation of E. coli biofilm formation. Moreover, this study provides a theoretical basis and feasibility reference for the application of optogenetics technology in biofilm-based immobilized fermentation.

## RESULTS

### Blue light signaling inhibits motility, curli formation, and biofilm formation in E. coli W1688.

**FIG 1:**
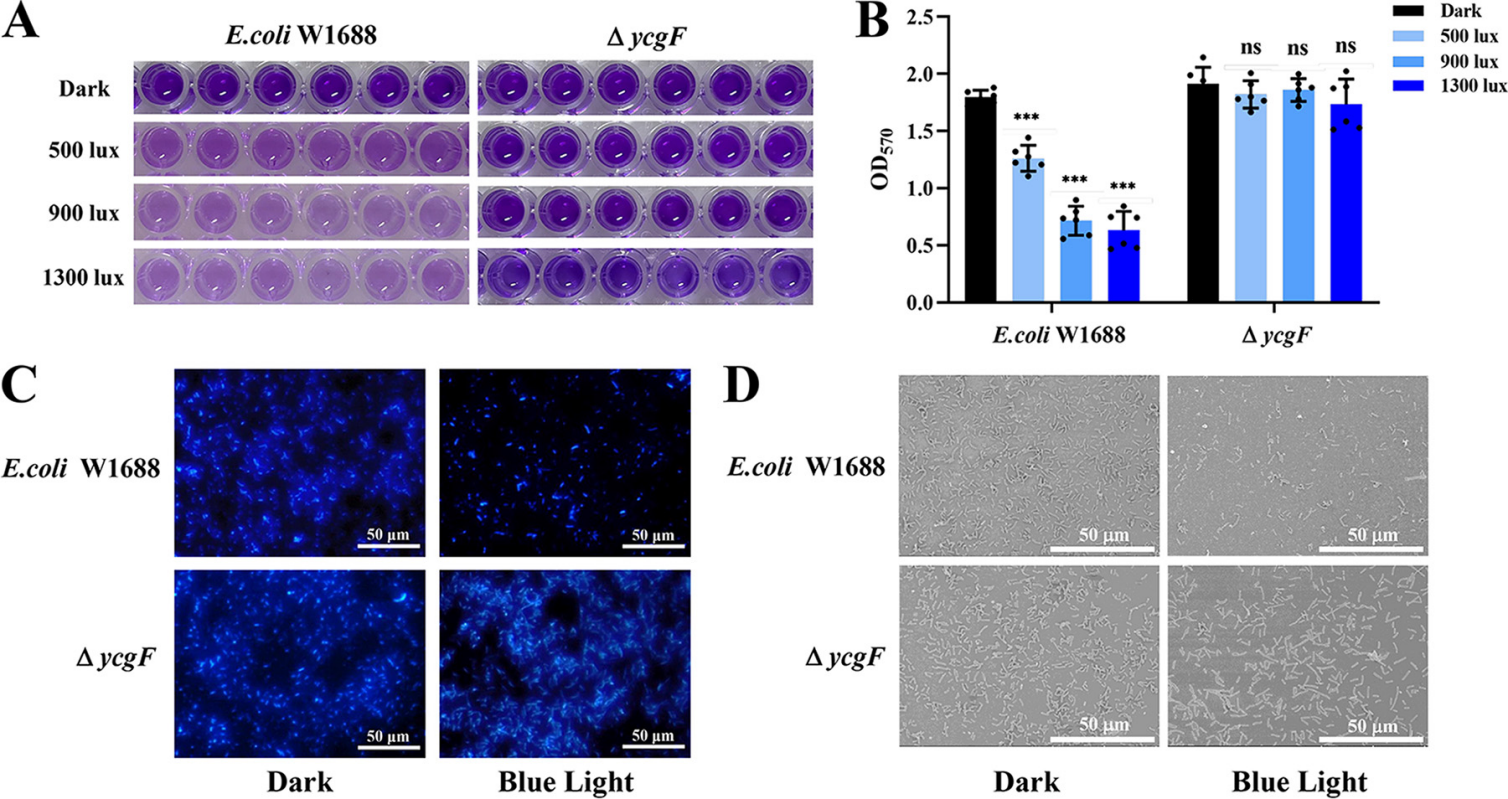
Effects of blue light on biofilm formation in E. coli. (A) E. coli W1688 and Δ*ycgF* strains were inoculated into a 96-well plate, incubated at 37°C under dark or blue light conditions with different light intensities for 24 h, and then imaged after crystal violet (CV) staining. (B) Corresponding OD_570_ value in the 96-well plate. (C) E. coli W1688 and Δ*ycgF* strains were incubated in 24-well plates with round coverslips under light or dark conditions. The biofilm cells on the coverslips were stained with DAPI, and the state of biofilm formation was observed under a fluorescence microscope. Scale bars, 50 μm. (D) Biofilm cells on the coverslips were subjected to ethanol gradient dehydration and observed via SEM. Scale bars, 50 μm. The values represent the means and standard deviations from three independent experiments. ***, *P* > 0.001; **, *P* > 0.01; and *, *P* > 0.05, by two-way ANOVA.

Light signaling regulates the lifestyles of nonphotosynthetic organisms (28). Our previous study also demonstrated that light signaling regulates the formation of Aspergillus niger biofilm (32). To evaluate the effect of blue light signaling on E. coli biofilm formation, semiquantitative experiments with crystal violet (CV) staining were performed on the biofilm of E. coli W1688 cells grown in 96-well plates. Comparing the biofilm formation ability of E. coli W1688 grown under different blue light intensities or dark conditions, the results of the CV assays were noted to differ significantly (Fig. 1A and B). With an increase in blue light intensity, the extent of staining gradually decreased in E. coli W1688 cells grown under blue light conditions compared with those grown under dark conditions; in addition, the optical density at 570 nm (OD_570_) value gradually decreased for E. coli W1688 cells grown under blue light conditions. When the blue light intensity was 1,300 lx, E. coli W1688 biofilm formation decreased by 64.81%. To further confirm the inhibitory effect of blue light signaling on E. coli W1688 biofilm formation, we performed 4′,6-diamidino-2-phenylindole (DAPI) staining and scanning electron microscopy (SEM) analysis to observe the changes in biofilm formation (Fig. 1C and D). E. coli W1688 cells grown under dark conditions appeared as a sheet in the microscopic field of view, whereas those grown under blue light conditions appeared as only scattered cells, and the cell density of the latter was significantly reduced compared with that of the former. Based on the above-mentioned results, it can be concluded that the blue light signaling inhibits E. coli W1688 biofilm formation.

**FIG 2:**
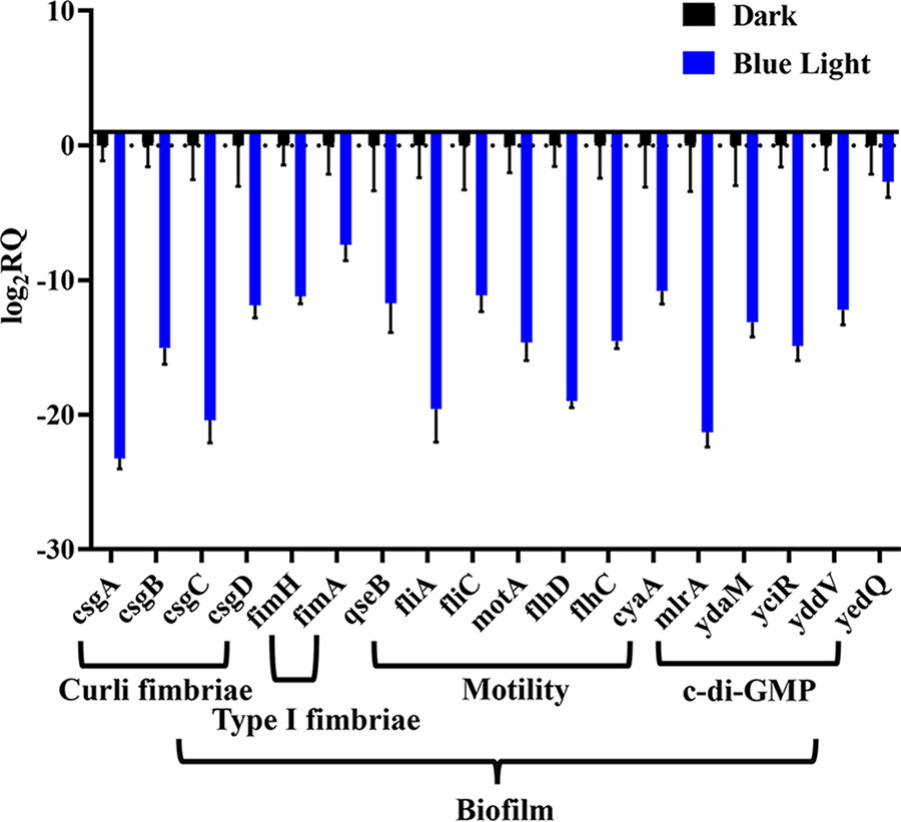
Decreased expression of biofilm-related genes under blue light conditions. Shown is expression of the genes involved in curli and type I fimbria formation, motility, and c-di-GMP level. The standard deviation of all gene expressions was *P* < 0.001. The values represent the means and standard deviations of values from three independent experiments. RQ, relative quantity. ***, *P* > 0.001; **, *P* > 0.01; and *, *P* > 0.05, by two-way ANOVA.

To further analyze the effect of blue light signaling on E. coli W1688 biofilm formation, quantitative reverse transcription-PCR (qRT-PCR) was performed targeting E. coli genes associated with biofilm formation. E. coli surface structures, c-di-GMP and autotransporter regulate biofilm formation by mediating cell-to-cell adhesion (33). The FIM family is involved in the regulation or formation of type I fimbriae (34), the CSG family is involved in the formation of curli (35), *qseB*, *motA*, *cyaA*, and the FLH and FLI families are involved in the regulation of the flagellum formation and bacterial motility (36), and *mlrA*, *ydaM*, *yciR*, *yddV*, and *yedQ* are associated with c-di-GMP levels in E. coli (37). The expression of these genes decreased significantly under blue light conditions (1,300 lx) (Fig. 2), which supports the observed phenotype of reduced biofilm formation in E. coli cells grown under blue light conditions. Furthermore, blue light signaling appears to affect the motility of E. coli W1688.

**FIG 3:**
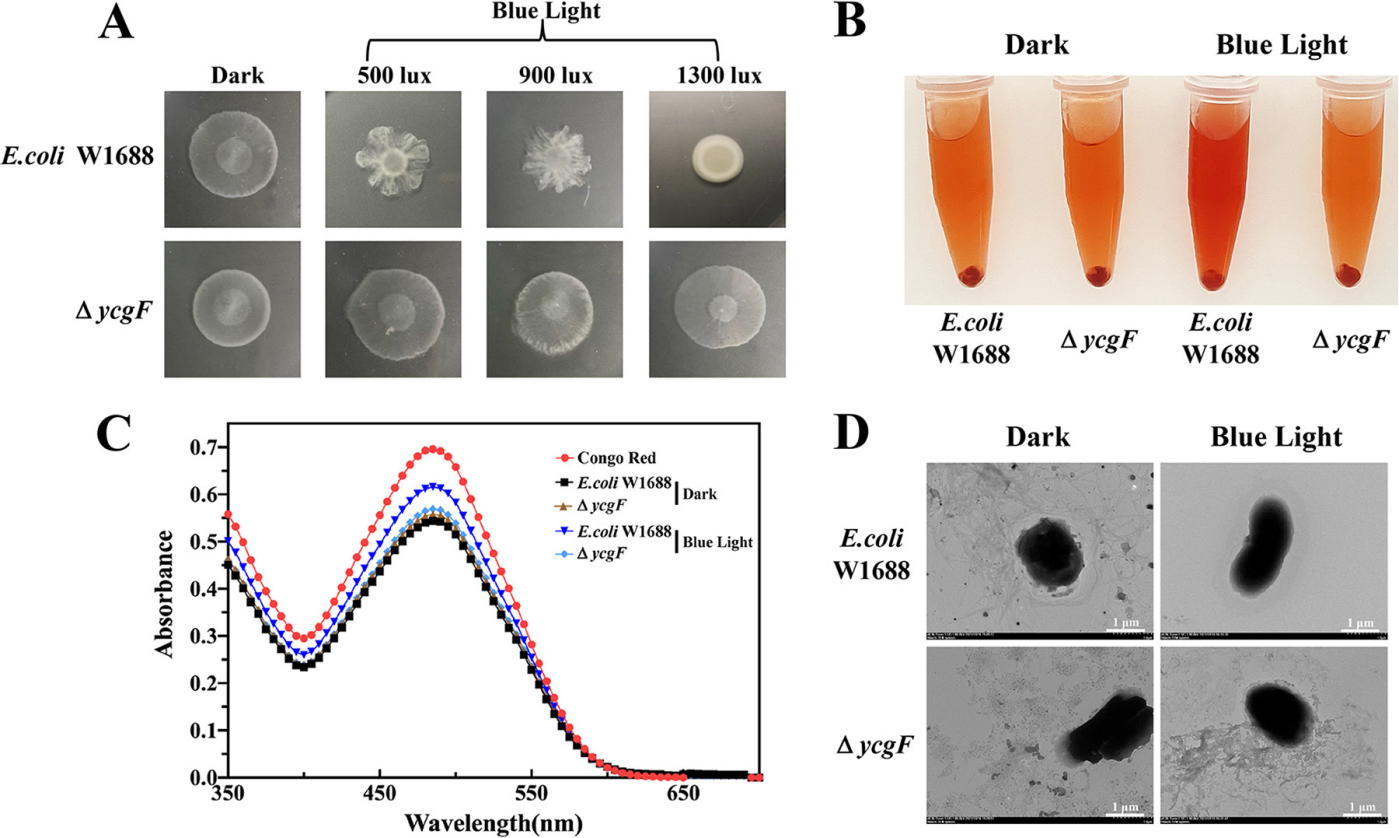
E. coli motility assay and curli fiber formation under dark or blue light conditions. (A) Chemotactic rings of E. coli W1688 and Δ*ycgF* strains observed on Eiken agar plates (diameter, 7 cm); (B) staining of E. coli curli fibers with Congo red (CR). (C) CR supernatant was used for full-wavelength analysis. (D) TEM images of E. coli W1688 and Δ*ycgF* strains. Scale bars, 1 μm.

Swarming motility is a rapid and coordinated migration of a bacterial population across a semisolid surface (38, 39). Therefore, the effect of blue light signaling on the motility of E. coli can be explored based on swarming. Based on our results (Fig. 3A and Table 1), the swarming diameter of E. coli W1688 was found to be significantly smaller in the strain grown under blue light conditions. With an increase in the blue light intensity, the bacterial circle gradually decreases. Compared with dark conditions, when the light intensity reached 500, 900, and 1,300 lx, there were 14.48%, 21.67%, and 32.45% reductions of the bacterial circle, respectively. Thus, blue light signaling appears to inhibit the swarming motility of E. coli W1688.

**TABLE 1:** Motility assay of E. coli W1688 and Δ*ycgF* mutant curli under dark or blue light conditions

Strain	Motility (diam in mm) under condition shown			
	Dark	Blue light		
		500 lx	900 lx	1,300 lx
E. coli W1688	20.03 ± 2.11	17.13 ± 2.84	15.69 ± 3.1	13.53 ± 1.35
Δ*ycgF* mutant	19.83 ± 4.5	23.08 ± 3.52	20.16 ± 1.94	22.47 ± 2.09

To further explore the effect of blue light signaling on the motility of E. coli W1688 and formation of curli fibers, E. coli W1688 was stained with Congo red (CR) and CR supernatant was measured at the full wavelength, and then the abundance of curli fibers was analyzed quantitatively. The extent of CR staining was evidently higher in strains grown under blue light conditions than in those grown under dark conditions (Fig. 3B). In addition, CR supernatant was used to measure the full wavelength. The absorbance of the CR supernatant of a sample grown under blue light conditions was significantly higher than that grown under dark conditions (Fig. 3C). To further verify the effect of blue light signaling on curli fibers, transmission electron microscopy (TEM) analysis was performed. E. coli W1688 cells grown under dark conditions exhibited a higher abundance of curli fibers and levels of extracellular secretions than those grown under blue light conditions (Fig. 3D). Thus, blue light signaling appears to inhibit the formation of E. coli W1688 curli fibers.

### Inhibitory effects of blue light signaling on biofilm formation are alleviated in YcgF-deficient strains.

Blue light signaling regulated the expression of genes and proteins related to downstream pathways through the blue light-sensing protein BluF (YcgF) in E. coli W1688. In addition, blue light signaling regulated the expression of structures/substances associated with biofilm, such as curli fibers and colanic acid, thereby affecting the motility and growth of the strain. Therefore, we constructed a strain lacking *ycgF* and explored the effects of blue light signaling on biofilm formation in this strain. First, biofilm formation was quantified using a CV assay. The biofilm formation ability of the Δ*ycgF* mutant grown under different blue light intensities or dark conditions was similar to that of E. coli W1688 grown under dark conditions; the OD_570_ value did not change significantly (Fig. 1A and B). E. coli W1688 and Δ*ycgF* strains grown under dark conditions stained more deeply than E. coli W1688 grown under blue light conditions, and their OD_570_ values were higher than that of the latter. In addition, changes were observed in E. coli W1688 biofilm formation and cell density after DAPI (4′,6-diamidino-2-phenylindole) staining and in SEM analysis (Fig. 1C and D). No significant differences in cell density were noted between E. coli W1688 and Δ*ycgF* strains grown under dark conditions and the Δ*ycgF* strain grown under blue light conditions. The above samples were at higher density than E. coli W1688 grown under blue light conditions, which supported the CV assay results. Based on the above-mentioned results, it can be concluded that the blue light signaling inhibits the biofilm formation of E. coli W1688 and YcgF regulates this effect. Therefore, YcgF plays an essential role in the regulation of E. coli W1688 biofilm formation under blue light conditions.

To investigate whether the deletion of *ycgF* alleviates the inhibition of E. coli W1688 motility, we performed a swarming assay. The results are shown in Fig. 3A and Table 1. The swarming diameters of the Δ*ycgF* strain grown under different blue light intensities (0, 500, 900, and 1,300 lx) were 99.00%, 115.23%, 100.15%, and 112.18% of those of E. coli W1688 grown under dark conditions. These swarming diameters did not vary considerably, and they are all larger than the swarming diameter of E. coli W1688 grown under blue light conditions. Because curli fibers regulate the motility of E. coli, CR staining and TEM analysis were performed. The E. coli W1688 and Δ*ycgF* strains grown under dark conditions as well as the Δ*ycgF* strain grown under blue light conditions were stained with CR similarly in centrifuge tubes (Fig. 3B). The absorbance values obtained by measuring the full wavelength using the CR supernatant were also similar (Fig. 3C). TEM analysis results showed that the abundance of curli fibers and levels of extracellular secretions were similar between Δ*ycgF* strains grown under blue light and dark conditions and E. coli W1688 grown under dark conditions but lower in E. coli W1688 grown under blue light conditions than those of the mutant strains (Fig. 3D). Thus, *ycgF* deletion appears to alleviate the inhibitory effects of blue light signaling on the motility of E. coli W1688.

**FIG 4:**
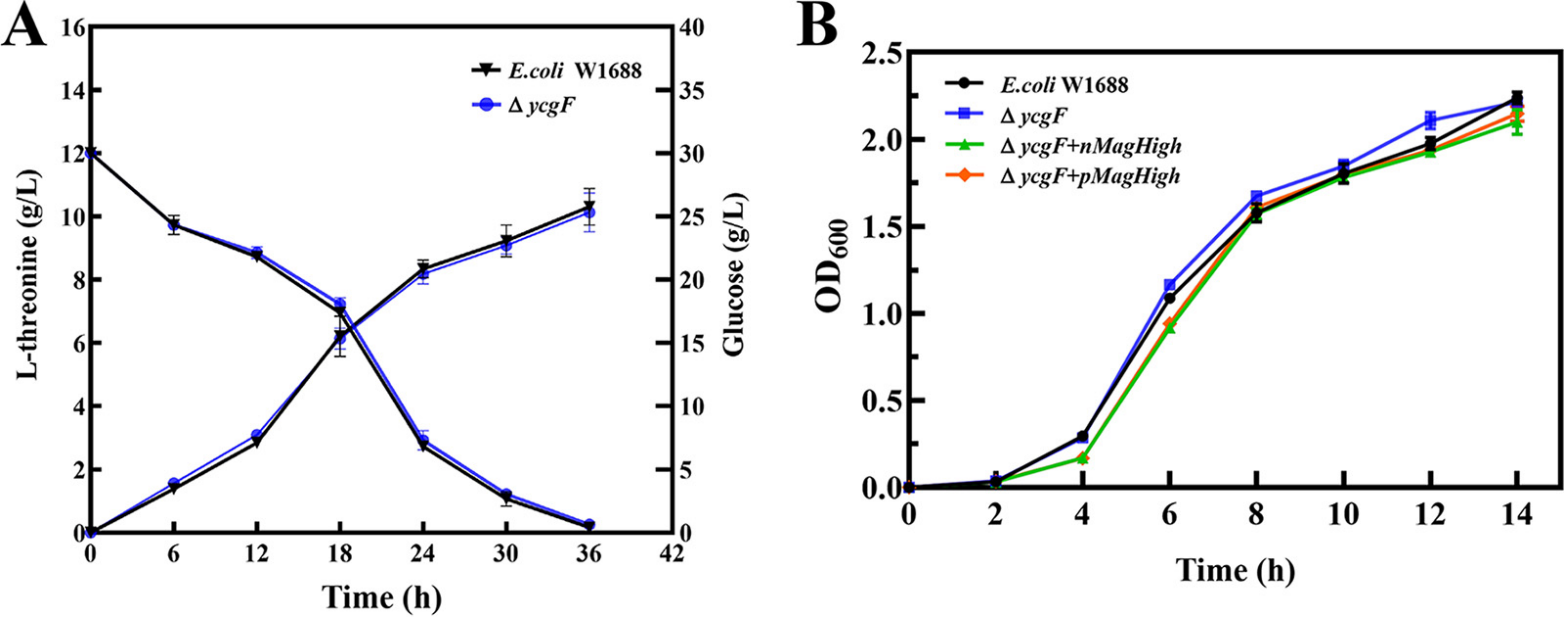
Free-cell fermentation and growth curves of E. coli W1688 and mutants. (A) l-Threonine production and glucose consumption in free-cell fermentation using E. coli W1688 and Δ*ycgF* strains. (B) Growth curves of E. coli W1688 and all mutants. The cell densities were determined by measuring OD_600_ at 2, 4, 6, 8, 10, 12, and 14 h.

To determine whether *ycgF* deletion affects fermentation performance, free-cell fermentation was performed using E. coli W1688 and Δ*ycgF* strains. The l-threonine yields of fermentation performed using E. coli W1688 and Δ*ycgF* strains was 10.31 and 10.13 g/L, respectively, after a single-batch fermentation. The l-threonine yields were similar for the two strains, and the fermentation periods were nearly the same (36 h) (Fig. 4A). Therefore, a platform strain that can use blue light as a regulation method was constructed.

### Magnet system promotes adsorption and biofilm formation in E. coli W1688.

To determine whether the deletion of *ycgF* and overexpression of the photosensitive Magnet system pMagHigh/nMagHigh affect the growth of E. coli W1688, growth curves were obtained at 2, 4, 6, 8, 10, 12, and 14 h. The OD_600_ values of the E. coli W1688, Δ*ycgF*, Δ*ycgF nMagHigh*, and Δ*ycgF pMagHigh* strains increased most rapidly at 4 to 8 h; all reached the peak at 14 h, with an OD_600_ value close to 2.0 (Fig. 4B). The growth trends were nearly the same, indicating that the deletion of *ycgF* and overexpression of pMagHigh or nMagHigh did not affect growth. Thus, the optogenetic system Magnet can be explored further in the Δ*ycgF* strain lacking a blue light-sensing protein.

**FIG 5:**
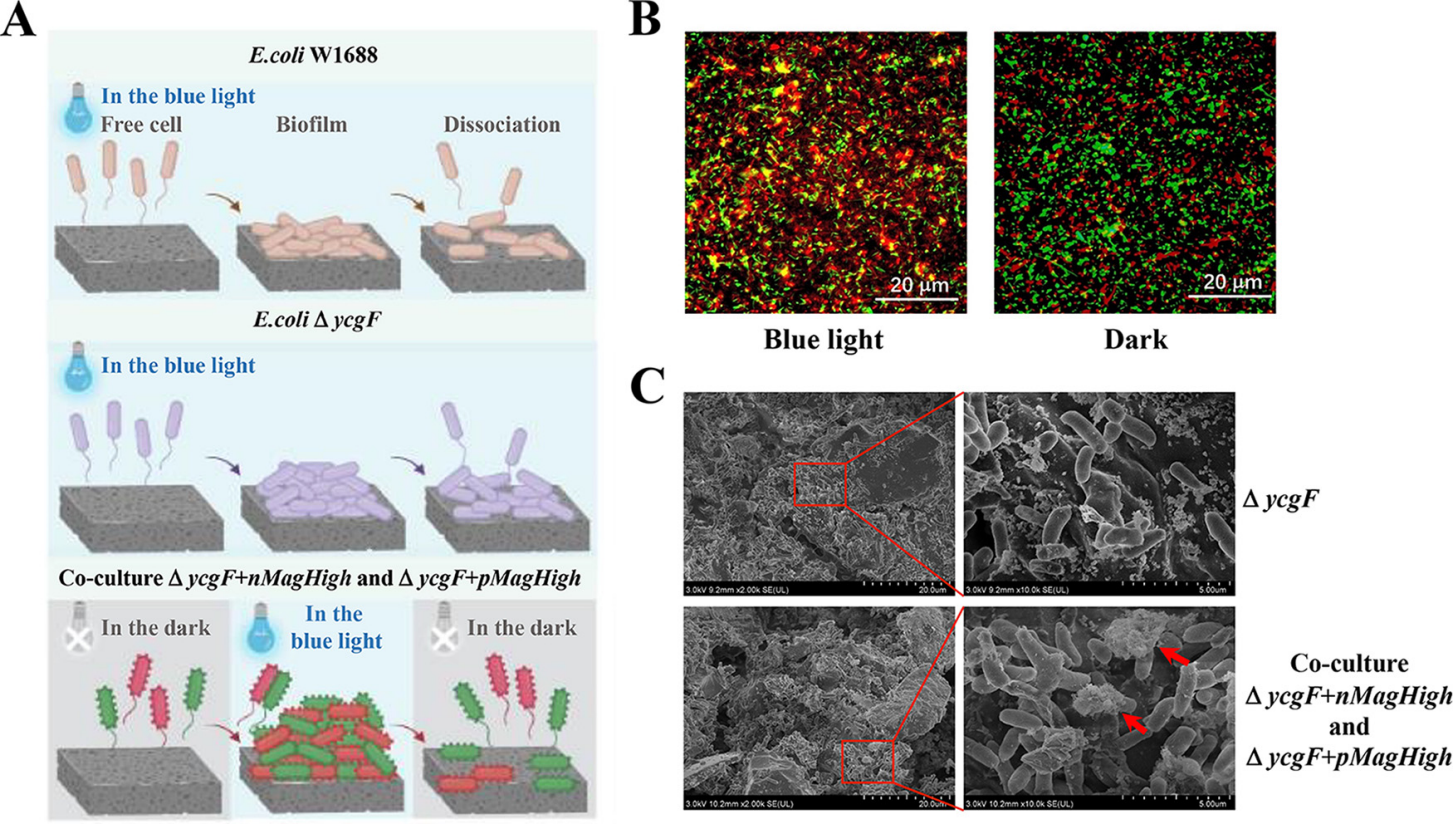
Adsorption and adhesion of the optogenetic system Magnet. (A) Biofilm formation in the E. coli W1688, Δ*ycgF*, Δ*ycgF nMagHigh*, and Δ*ycgF pMagHigh* strains; (B) blue light-dependent aggregation of bacteria expressing nMagHigh and pMagHigh. E. coli cells exhibiting nMagHigh (labeled with EGFP) or pMagHigh (labeled with mCherry) were mixed in a 1:1 ratio (OD_600_ = 0.15) and incubated for 4 h under blue light or dark conditions. Scale bars, 20 μm. (C) SEM analysis of sample obtained from immobilized continuous fermentation using the Δ*ycgF* strain and the coculture of Δ*ycgF nMagHigh* and Δ*ycgF pMagHigh* strains.

The Magnet system effectively regulated the formation of E. coli biofilm via the induction of blue light signaling (Fig. 5A). By coculturing two double-expression strains (expressing nMagHigh and enhanced green fluorescent protein [EGFP] and pMagHigh and mCherry), aggregation and biofilm formation abilities of E. coli W1688 were observed through the expression of red-green fluorescent protein under a fluorescence microscope. Under dark conditions, the cells containing mCherry and EGFP were in a dispersed state and were distinct. However, E. coli cells aggregated into clusters under blue light conditions. In addition, mCherry and EGFP were aggregated and superimposed to form a relatively intuitive yellow cluster, but no such effect was noted under dark conditions (Fig. 5B).

To analyze whether the Magnet system modulated the rate of biofilm formation on carriers during immobilised fermentation, SEM analysis was performed. SEM results are shown in Fig. 5C. The Δ*ycgF* cells were relatively dispersed and extracellular matrix was relatively less under blue light conditions. However, the coculture of Δ*ycgF nMagHigh* and Δ*ycgF pMagHigh* strains generated a substantial amount of extracellular matrix to make the cells adhere together, aggregate into clusters, and form more biofilms under blue light conditions. Based on the above-mentioned results, the coculture of Δ*ycgF nMagHigh* and Δ*ycgF pMagHigh* strains appears to enhance biofilm formation under blue light conditions.

### Magnet system promotes the production of l-threonine in immobilized fermentation using E. coli W1688.

**FIG 6:**
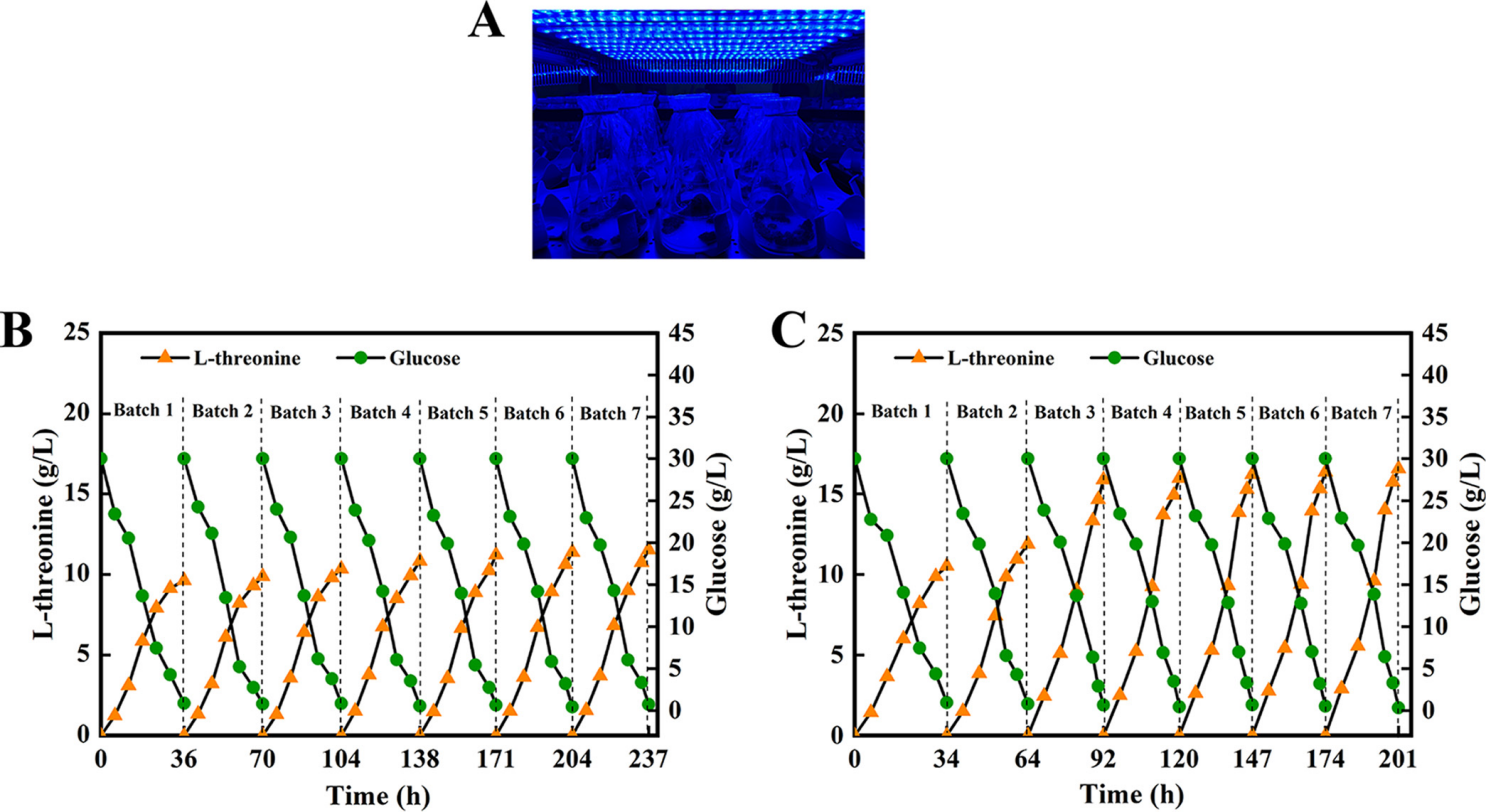
l-Threonine production and glucose consumption in immobilized continuous fermentation using Δ*ycgF* strain and the coculture of Δ*ycgF nMagHigh* and Δ*ycgF pMagHigh* strains. (A and B) Outcomes of immobilized continuous fermentation using (A) the Δ*ycgF* strain and (B) the coculture of Δ*ycgF nMagHigh* and Δ*ycgF pMagHigh* strains; (C) schematic representation of a blue light oscillation incubator in operation.

To further improve the fermentation efficiency, a polyurethane-based carrier was used for immobilized continuous fermentation under blue light conditions (Fig. 6A). During immobilized continuous fermentation, l-threonine yield gradually increased in the first four batches of fermentation using Δ*ycgF* strain and became stable in the fourth batch. The final fermentation yield was 11.54 g/L, and the fermentation period was shortened from 36 to 33 h (Fig. 6B). When Δ*ycgF nMagHigh* and Δ*ycgF pMagHigh* strains were cocultured under blue light conditions, the l-threonine yield of the first two batches of fermentation increased gradually and that of the third batch of fermentation increased significantly and gradually became stable. The final fermentation yield was 16.57 g/L, and the fermentation period was shortened to 27 h, which was 43.59% higher than that of immobilized continuous fermentation using the Δ*ycgF* strain (Fig. 6C). The yield of fermentation using the coculture of Δ*ycgF nMagHigh* and Δ*ycgF pMagHigh* strains reached 11.89 g/L in the second batch of fermentation, which was similar to the seventh batch of immobilized continuous fermentation using the Δ*ycgF* strain and even exceeded the final yield of fermentation using the Δ*ycgF* strain. Compared with the Δ*ycgF* strain, the coculture of Δ*ycgF nMagHigh* and Δ*ycgF pMagHigh* strains increased the yield of immobilized continuous fermentation, shortened fermentation time, accelerated fermentation, and improved fermentation efficiency. Even the fermentation level of the Δ*ycgF* strain can be reached in a relatively short time. In addition, as illustrated in Table 2, the use of the Magnet system in l-threonine production has certain advantages over the use of industrial E. coli strains, which have been used to produce l-threonine in recent years.

**TABLE 2:** Comparison of fermentation performance of different E. coli engineered strains

Method and strain	Time (h)	l-Threonine (g/L)	Productivity (g/L/h)	Reference
Free-cell fermentation				
W1688	36	10.3	0.2861	This study
Δ*ycgF* mutant	36	10.12	0.2811	This study
Batch/shake flask culture				
Δ*ycgF* mutant	33	11.54	0.35	This study
Δ*ycgF nMagHigh* and Δ*ycgF pMagHigh* mutants cocultured	27	16.57	0.614	This study
TWF006/pFW01	36	15.9	0.44	50
βIM4	72	13.4	0.186	51
Fed-batch culture				
TH28C	50	82.4	1.648	52
EC125	48	105.3	2.194	53
THPE5	40	70.8	1.77	54
TWF083	48	116.62	2.43	55

## DISCUSSION

Biofilms play an essential role in the environment, industry, medicine, and other sectors. Currently, exploring the mechanism of biofilm formation has become a research hot spot. In the context of industrial manufacturing, biofilms have high resistance and activity, which help microorganisms to produce biochemicals efficiently. However, limited studies have focused on industrial E. coli biofilm formation; thus, directionally regulating and increasing the rate of biofilm formation appear to be crucial. Recent research has focused on the application of various optogenetic techniques in model organisms, and using optogenetics to regulate biofilm formation represents a new approach for the use of these techniques (40). Surprisingly, we found that blue light signaling inhibited E. coli biofilm formation in study process. Therefore, in this study, we investigated the mechanism underlying the inhibitory effects of blue light signaling on E. coli W1688 biofilm, combined with the optogenetic component Magnet system to further analyze its biofilm formation in this strain under blue light conditions and predict the feasibility in industrial fermentation.

**FIG 7:**
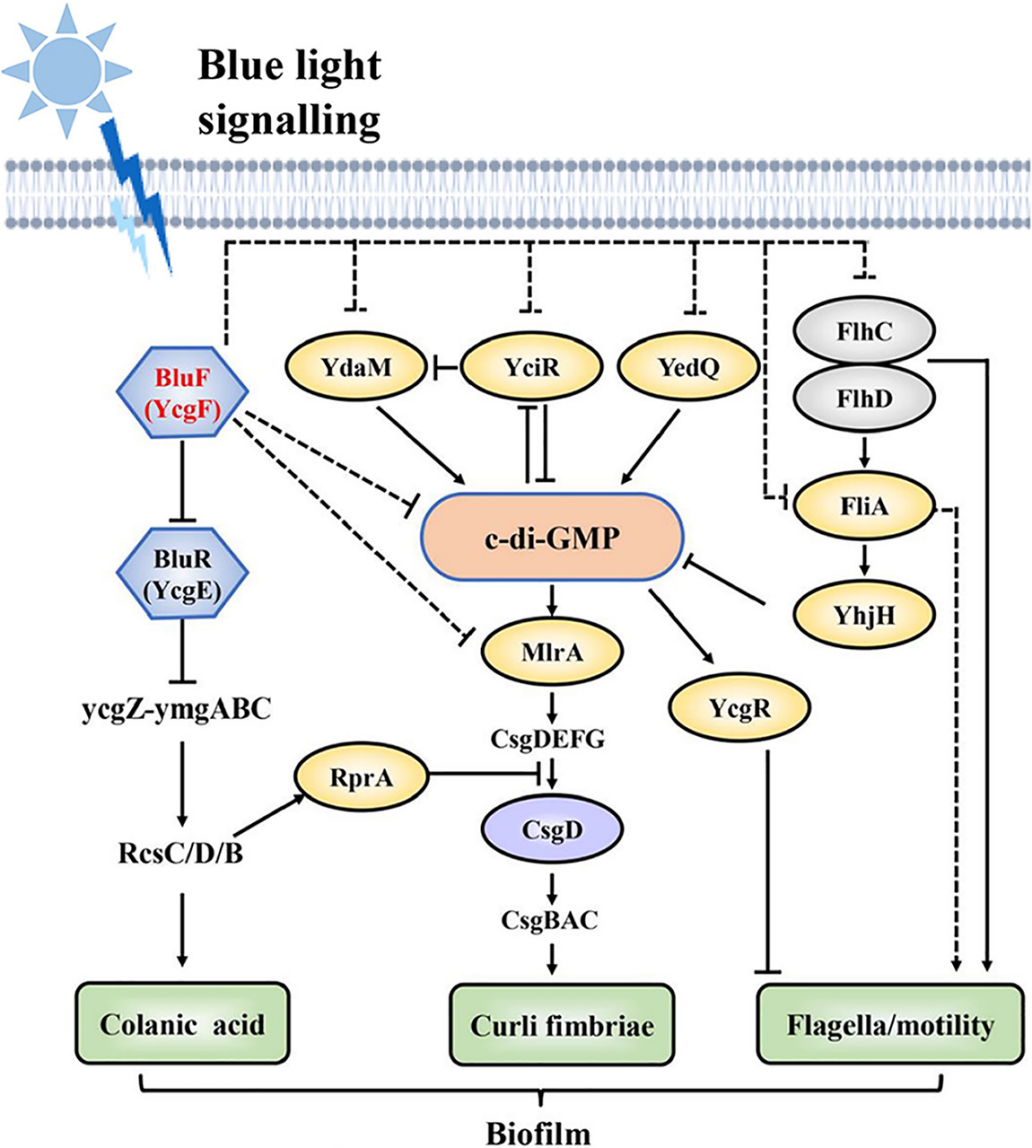
E. coli senses blue light via the BluF-EAL protein BluF (YcgF), which controls various functions of biofilm. Solid arrows show activation or inhibition, and dotted arrows show an indirect effect.

E. coli regulates the expression of genes and proteins associated with downstream pathways through the blue light-sensing protein BluF (YcgF). YcgF directly antagonizes the MerR-like repressor BluR (YcgE), which leads to expression of the *ycgZ*-*ymgABC* operon and activation of the Rcs system. Then YcgF regulates the expression of biofilm formation-related structures/substances, such as c-di-GMP, curli fibers, and colanic acid, which affect the biofilm’s motility and formation (Fig. 7). We constructed a strain lacking a blue light-sensing protein, the Δ*ycgF* mutant, to determine whether the blue light signaling affects the growth and maturity of biofilm in E. coli W1688. The biofilm of E. coli W1688 was qualitatively and quantitatively analyzed via CV and DAPI staining as well as SEM analysis; blue light signaling inhibited the formation of E. coli W1688 biofilm. The biofilm formation-related signaling pathways of E. coli W1688 grown under blue light conditions were analyzed through qRT-PCR. Moreover, the results of swarming analysis and CR staining confirmed that blue light signaling inhibited the curli fiber formation and motility of E. coli W1688. Together, these results suggest that blue light signaling inhibits curli fiber formation, motility, and biofilm formation in E. coli W1688. Furthermore, *ycgF* deletion alleviated the inhibitory effects of blue light signaling on E. coli W1688 biofilm formation and motility to a certain extent.

As an industrialized strain, the ultimate purpose of the modification of E. coli is its application to the industrial production of l-threonine. Therefore, we conducted a preliminary study on the growth ability of this strain. The growth state of modified strains is mostly the same as that of E. coli W1688. A single-batch free-cell fermentation experiment was performed using E. coli W1688 and Δ*ycgF* strains; the fermentation abilities were similar between these two strains. Consequently, we speculate that the modification of these genes exerts no effect on the growth and fermentation ability of the strains used in the experiment and a platform strain that can use blue light as a regulatory method was successfully constructed.

Notably, the optogenetic elements nMagHigh and pMagHigh act as adhesins. They heterodimerize under the blue light conditions (450 nm), attract each other, and aggregate into clusters; on the other hand, these elements dissociate from each other under dark conditions, thereby regulating biofilm formation (23, 26, 41). Owing to the induction of the blue light signaling, the two overexpressed strains aggregated into clusters, forming a more intuitive yellow colony and biofilm. In 1971, Marshall et al. (42) reported using SEM analysis that attached bacteria are associated with the surface via fine extracellular polymeric fibrils. The results of SEM analysis performed in our study showed that after the carriers containing the Magnet system were treated with blue light, a large amount of extracellular matrix was generated to facilitate bacterial adherence and thus biofilm formation. The above-mentioned results confirm that due to the use of the blue light signaling-induced Magnet system, the Δ*ycgF nMagHigh* and Δ*ycgF pMagHigh* strains, which were originally distributed irregularly, attracted each other and gathered into clusters. Compared with the Δ*ycgF* strain, the biofilm production efficiency of the Δ*ycgF nMagHigh* and Δ*ycgF pMagHigh* strains was improved, which laid a foundation and serves as a reference for the subsequent immobilized continuous fermentation for l-threonine production.

Finally, we attempted to determine whether the application of optogenetics improves the l-threonine-production efficiency of immobilized continuous fermentation. When the blue light intensity is high, the morphology of E. coli cells may change; however, whether the morphology change affects the production efficiency remains unknown (43). To evaluate the l-threonine production efficiency, a lower intensity of blue light (500 lx) was selected during immobilized fermentation. (Under this blue light intensity, the Magnet system can be used without altering E. coli morphology.) The yield of second batch of immobilized continuous fermentation using the coculture of Δ*ycgF nMagHigh* and Δ*ycgF pMagHigh* strains matched the yield of the seventh batch of fermentation using the Δ*ycgF* strain. Using the Magnet photosensitive system in the immobilized continuous fermentation using E. coli not only shortens the fermentation period and improves the yield but also shortens the adsorption process. Moreover, the operation is simple and convenient, a high degree of control of time and space is achieved, and the utility model has a satisfactory application prospect.

Based on current research, we sought to detect the content of E. coli biofilm formation-related signaling molecules under blue light and dark conditions and then improve the signaling pathway of blue light signaling on E. coli biofilm formation. We further developed and used the EL222 activation system to ensure the directional regulation of blue light signaling and improve the production efficiency of the biochemical product l-threonine. Using the EL222 activation system, the expression of the Magnet system or key genes in biofilm can be regulated in a real-time controllable expression (44). Rational use of key components is helpful for light-controlled directional regulation of E. coli biofilm formation, which reduces the biofilm formation pressure, promotes glucose conversion rate and l-threonine yield, and shortens the fermentation period. All these topics will be explored in follow-up studies.

In conclusion, blue light signaling regulates E. coli biofilm formation through YcgF. YcgF regulates the curli fiber formation in E. coli grown under blue light conditions and affects the motility of this organism. Through a series of biofilm phenotype-related experiments performed using E. coli W1688 grown under blue light conditions, we verified the inhibitory effect of blue light on E. coli biofilm formation. Furthermore, our Δ*ycgF* mutant strain represents a platform strain that can use blue light as a regulatory method. This strain was further combined with the Magnet system to obtain two regulatable biofilm-enhancing strains. Among them, in cocultures, the Δ*ycgF nMagHigh* and Δ*ycgF pMagHigh* strains exerted significant effects on the shortening of fermentation cycle, reducing fermentation batch numbers and improving fermentation efficiency in l-threonine production. Therefore, these strains should be used in industrial production to increase l-threonine yield and alleviate the pressure associated with high yield in industrial production. This study opens up new avenues and lays the foundation for using other microorganisms with optogenetic elements.

## MATERIALS AND METHODS

### Strains and media.

All strains, plasmids, and growth conditions used in this study are shown in Table 3. E. coli W1688 producing l-threonine (the preservation number is CCTCC M2015233) was obtained from E. coli MG1655 (ATCC 47076) through molecular modification. E. coli W1688 and its transformed strain were grown in Luria-Bertani (LB) medium at 37°C. The fermentation medium was prepared as described previously (14). The medium contained the following concentrations of appropriate antibiotics: 50 mg/L kanamycin (Kan) and 40/mg/L streptomycin (Str). The following concentrations of appropriate induction reagents were used for induction: 0.5 mM isopropyl-β-d-1-thiogalactopyranoside (IPTG) and 30 mM l-arabinose. The strain was grown and fermented at 37°C and 200 rpm.

**TABLE 3:** E. coli strains and plasmids used in this study

Strain or plasmid	Description	Source
Strains		
W1688	l-Threonine-producing strain from E. coli MG1655 by mutation and molecular modification	CCTCC M2015233
Δ*ycgF* mutant	E. coli W1688 with deletion of *ycgF*	This study
Δ*ycgF nMagHigh* mutant	Δ*ycgF* strain harboring plasmid pET28a-EGFP-nMagHigh-OmpX	This study
Δ*ycgF pMagHigh* mutant	Δ*ycgF* strain harboring plasmid pET28a-mCherry-pMagHigh-OmpX	This study
T1		This study
Plasmids		
pET28a-EGFP	Kan^r^, T7 promoter + EGFP	This study
pET28a-EGFP-nMagHigh	Kan^r^, T7 promoter + EGFP, trc promoter + nMagHigh-OmpX	This study
pET28a-mCherry	Kan^r^, T7 promoter + Cherry	This study
pET28a-mCherry-pMagHigh	Kan^r^, T7 promoter + mCherry, trc promoter + pMagHigh-OmpX	This study
pCas	Kan^r^, 30°C	This study
pTarget	AadA^r^, 37°C	This study

Illumination for gene expression, fermentation, and optogenetics applications was performed using a blue LED panel (a square light source containing 270 blue LED bulbs) placed in a shaking incubator (Shanghai Zhichu Instrument Co., Ltd.; ZQZY-88BGN) (60 by 55 by 40 cm^3^) at a distance of 10 cm from the cell culture. The light intensity can be adjusted using a switching power supply with a controller and adjusting the distance of the blue LED panel from the cell culture. The blue light intensity used in this study was 500 to 1,300 lx.

### Δ*ycgF*, Δ*ycgF nMagHigh*, and Δ*ycgF pMagHigh* strain construction.

The Δ*ycgF* strain was obtained from the E. coli W1688 strain by knocking out the target gene *ycgF* using the CRISPR/Cas9 gene-editing technology. The primers used are shown in Table S1 in the supplemental material. The specific operation is as follows. The donor DNA fragment and pTarget plasmid containing the *N*_20_ sequence were constructed and introduced into competent E. coli W1688 cells containing the pCas plasmid. Using Kan (50 mg/L) and Str (40 mg/L) as resistance markers, the transformed cells were screened to select the cells lacking *ycgF*.

To express nMagHigh and pMagHigh on the surface of E. coli, we fused these proteins to the N terminus of OmpX (45). To insert the *nMagHigh* gene into the plasmid pET28a, the gene cassettes *nMagHigh*-linker-OmpX and *pMagHigh*-linker-OmpX were first synthesized by Tsingke Biotechnology Co., Ltd. Then, using the ClonExpress II one-step cloning kit C112-01 (Vazyme Biotech Co., Ltd.), these genes were inserted into the BglII restriction sites of pET28a-EGFP and pET28a-mCherry, respectively. Thus, plasmids pET28a-EGFP-nMagHigh-OmpX and pET28a-mCherry-pMagHigh-OmpX were obtained. The constructed plasmid was transformed into Δ*ycgF* using the heat shock transformation method, and the transformants were selected using Kan (50 mg/L).

### Growth curve.

Bacterial biomass was determined by measuring absorbance at a wavelength of 600 nm (1). The four strains after overnight culture were diluted to obtain an OD_600_ value of 0.1 and inoculated into fresh LB medium. The nMagHigh and pMagHigh mutant strains were induced by adding Kan (50 mg/L) and IPTG (0.5 mM), respectively. Sampling was performed at 2, 4, 6, 8, 10, 12, and 14 h, the OD_600_ of the samples was measured using a spectrophotometer, and the growth curves of the four strains were drawn.

### Biofilm formation assay.

CV is a basic dye that can help quantify the extent of biofilm formation by binding and staining with negatively charged surface molecules and cells, proteins, polysaccharides, and other substances (46–48). The E. coli W1688 and Δ*ycgF* strains were cultured until an OD_600_ value of 1 was recorded, and 20 μL bacterial suspension was added to a 96-well microtiter plate containing 180 μL fresh LB medium. Bacterial cultures were incubated under dark or blue light (450 nm; 500 to 1,300 lx) conditions at 37°C for 24 h. Each sample was washed with phosphate-buffered solution (PBS); next, the samples fixed with 4% paraformaldehyde (PFA) (200 μL), and then 1% CV (200 μL) was added to stain the biofilm. After washing and drying, an acetic acid solution was added to dissolve the CV. Biofilms were quantified by measuring the absorbance of the samples at 570 nm using a spectrophotometer. At least eight independent repeats were performed for each strain.

In some cases, cell slides were used as a vehicle to observe biofilms under a microscope. The DNA-specific probe DAPI stains cells with intact cell membranes, thereby helping to monitor biofilm density via fluorescence microscopy (48); it was used to explore changes in the biofilm formation of E. coli W1688 and Δ*ycgF* strains. The two strains were cultured until an OD_600_ value of 1 was recorded, and 200 μL bacterial suspension was added to a 24-well plate containing 1,800 μL fresh LB medium. The bacterial cultures were incubated under dark or blue light (450 nm; 1,300 lx) conditions at 37°C for 30 h. After the culture, the cell slides were taken out, 4% PFA was added, the cells were fixed on the cell slides, excess fixative was washed off, DAPI staining solution was added for staining, and the results were observed under a fluorescence microscope.

SEM analysis facilitates detailed and high-resolution three-dimensional visualization of biofilms, allowing cell-climbing sheets as bacterial carriers to observe biofilm structure and function (48). E. coli W1688 and Δ*ycgF* strains were cultured in cell slides following the above-mentioned culture method. After the culture, the slides were taken out and washed three times with PBS, and then the samples were dehydrated using a gradient of 50%, 70%, 80%, and 90% ethanol. After the samples were dried, they were plated with gold film with a Hitachi E-1010 ion sputter coater and then placed under a SU8010 field emission scanning electron microscope for observation and imaging.

### Mobility analysis.

The swarming plate medium contained 20 g/L NaCl, 20 g/L peptone, 10 g/L yeast extract, 3 g/L glucose, and 3 g/L Eiken agar (Eiken Chemical, Japan). Overnight-cultured strains (2.5 μL) were inoculated into the center of the swarming plate and incubated at 37°C for 24 h under dark and blue light (450 nm; 500 to 1,300 lx) conditions to observe bacterial growth on the swarming plate.

### CR stain and TEM.

CR is a diazo textile dye used for the qualitative analysis of cellulose and amyloid fibers as well as curli fibers produced by E. coli. It is also used in indirect identification measurement of c-di-GMP (48, 49). E. coli W1688 and Δ*ycgF* cell suspension samples were stained with 5 μL CR (25 mg/mL), which was followed by full-wavelength scanning to quantitatively analyze the amount of CR on the biofilm and further determine the amount of biofilm. TEM analysis facilitates high-resolution visualization of specific structural components, such as polysaccharide fibrils and environmental DNA (eDNA) (48). The two strains were cultured until an OD_600_ value of 1 was recorded and then added to a 24-well plate containing fresh M63 medium and incubated under dark and blue light (450 nm; 1,300 lx) conditions for static culture at 30°C for 30 h. The bacteria were collected via centrifugation (12,000 × *g*, 2 min), resuspended, and washed with 1% PBS to prepare suspension samples. The suspension sample was dropped on a copper mesh with a support membrane and retained for 2 min; the excess liquid was absorbed from the edge of the droplet with filter paper. The copper mesh was inserted into the phosphotungstic acid dye solution and dyed for 15 s. After drying, the samples were observed under the Hitachi H-7700 TEM system.

### Carrier preprocessing.

Using polyurethane as a raw material, a new type of porous foam carrier was prepared in our laboratory, which was named S7. The size of the carrier was reduced to 10 by 10 by 10 mm^3^. It was soaked in an appropriate volume of 1 M NaOH for 1 h and washed with ultrapure water. Then, it was soaked in 1 M HCl solution for 1 h, washed with sterile water to obtain a pH value of 7.0, and put into an oven to dry the water. An amount of 30 g/L S7 was added to the fermentation broth, which was sterilized at 115°C for 20 min and then cooled.

### Free-cell fermentation, immobilized fermentation, and carrier SEM analysis.

E. coli W1688 and Δ*ycgF* strains were cultured at 37°C and 200 rpm to the logarithmic growth phase of the strains and then inoculated into LB liquid medium at an inoculum size of 5%; next, the corresponding resistance was added and induced for 12 h following the above-mentioned culture method. Then, the strains were added to the fermentation medium at an inoculum size of 2% and fermented at 37°C and 200 rpm; sampling was performed every 6 h. The samples were centrifuged at 4°C and 12,000 × *g* for 5 min, and the supernatant was collected to measure the levels of l-threonine and glucose. Sampling was stopped when the glucose in the fermentation broth was exhausted.

Using the same fermentation broth as free-cell fermentation, immobilized continuous fermentation was performed using Δ*ycgF* strain and the coculture of Δ*ycgF nMagHigh* and Δ*ycgF pMagHigh* strains. Based on free-cell fermentation culture conditions, blue light (450 nm; 500 lx) was used for irradiation and 30 g/L of the carrier was added to the fermentation broth for immobilized fermentation. When the glucose level in the fermentation broth was lower than 0.1 g/L, the first batch of fermentation ended and the next batch of fermentation began. After each fermentation batch, part of the fermentation broth was aspirated from the container, the carrier was retained, and an equal volume of sterile fermentation broth was added for the next batch of fermentation. The immobilized fermentation was stopped until the l-threonine production was stable.

The carriers were then taken out and soaked in PBS: 2.5% glutaraldehyde was added to them, they were fixed at 4°C for 12 h, and then excess fixative solution was absorbed. Afterwards, 0%, 50%, 70%, 80%, 90%, 95%, and 100% ethanol were used for gradient dehydration, and each concentration of alcohol was dehydrated twice for 15 min each time. Then, the samples were soaked in *tert*-butanol for 10 min, centrifuged to remove *tert*-butanol, and placed in a −80°C refrigerator overnight. Finally, the samples were freeze-dried using a vacuum lyophilizer and sent for SEM analysis via Hitachi SU8020.

### Bacterial aggregation assay.

The E. coli Δ*ycgF nMagHigh* (tagged with EGFP) and Δ*ycgF pMagHigh* (tagged with mCherry) overexpression strains were mixed in a ratio of 1:1 for analysis (OD_600_ = 0.15). Each bacterial culture (300 μL) was added to a 24-well plate and incubated under the static condition for 4 h under dark or blue light (450 nm; 1,300 lx) conditions. Next, 200 μL of 10% glutaraldehyde fixative was added to each sample for 30 min before fluorescence microscopy images were obtained.

### qRT-PCR analysis.

To a 24-well plate containing 1,800 μL of fresh LB medium, 200 μL of E. coli W1688 bacterial solution (OD_600_ = 1) was added; then the culture was incubated at 37°C for 24 h under dark or blue light (450 nm; 1,300 lx) conditions. The culture was then centrifuged at 4°C and 12,000 × *g* for 2 min, and the cell pellet and supernatant were separated. RNA extraction, cDNA preparation, and qRT-PCR analysis were performed as described previously (14). The genes and primers used for qRT-PCR are shown in Table S2. All experiments were performed at least three times. We used a false-discovery rate threshold of ≤0.001 and an absolute value of the log_2_ ratio of ≥1 as the criteria for assessing the significance of differential gene expression.

### Statistical analysis.

All experiments were performed at least three times. Statistical significance was analyzed using one- or two-way analysis of variance (ANOVA) or paired *t* test using the GraphPad Prism software (San Diego, CA, USA). Data are reported as the means and standard deviations of values from three independent experiments. *P* values were computed using Student's *t* test (n.s., not significant at *P* > 0.05; ***, *P* < 0.001; **, *P* < 0.01; *, *P* < 0.05).
