# Supplementary material for: Blue Light Signaling Regulates Escherichia coli W1688 Biofilm Formation and l-Threonine Production
Source: Microbiol Spectr. 2022 Sep 27;10(5):e02460-22. doi: 10.1128/spectrum.02460-22 (PMC9604211; doi:10.1128/spectrum.02460-22)
Supplement: Supplemental file 1 — Table S1 and Table S2. Download spectrum.02460-22-s0001.pdf, PDF file, 0.1 MB [file spectrum.02460-22-s0001.pdf]

Supplementary information

Table S1 Sequence of the oligonucleotide primers used for gene knockout and plasmid construction in this study.

Primer name	Primer sequence (5' to 3')
ycgF up-F	GTTTCTGTTCCTCTTCCCCAGTCG
ycgF up-R	TAACAATCCAGGGTAATGGGTGAGG
ycgF down-F	TTTTCAGCACATTCTTTCACATGATTTCAGT
ycgF down-R	CCAGTGAAATAATTCTCGCGCG
ycgF check-F	CCTGTTAAATAATCAGGCTGTTGCATT
ycgF check-R	CGCTGGCGAACAGGAAGGTA
nMagHigh/pMagHigh-linker-OmpX-F	TTGACAATTAATCATCCGGCTCGTATAATG
nMagHigh/pMagHigh-linker-OmpX-R	ATTTGTCCTACTCAGGAGAGCGTT

Table S2 Sequence of the oligonucleotide primers used for qRT-PCR in this study.

Gene	Forward primer sequence (5' to 3')	Reverse primer sequence (5' to 3')
16S RNA	TCGGGAACCGTGAGACAGG	CCGCTGGCAACAAAGGATAAG
mlrA	TTCGAAGGCCGTACATTGCT	GGCCTTGTTCTTGCCAACTG
ydaM	CGCGATGACCGGATTACTGA	GTAATCCTGAGCGATGGCGA
yciR	GTCATTGGGCAAAGCGTGTT	TGCATTGCCGCTTCGAAAAA

yddV	GTGATGACTCGCGCGTTTAC	TCGGCGTTTTCCAGTAACGA
yedQ	TGATCGCCAAGAGTACGACG	CGAATACCCGATTACGCCCA
csgA	GATCTGACCCAACGTGGCTT	GTCACGTTGACGGAGGAGTT
csgB	AGCCGCAGCAGGTTATGATT	CCTGCCGTAACTGAGCACTA
csgC	CCCAGCAAGGGGATGTGTAT	AGCAGGCAATGAAAGGGTCT
csgD	GATTACCCGTACCGCGACAT	CGTAAAGTAGCATTCGCCGC
fimH	TGGCGATTAAAGCTGGCTCA	CACGAGCAGAAACATCGCAG
fimA	CATCTAAAGCCGCTGTTGCC	GTCCAGGATCTGCACACCAA
qseB	ATTGGCGACGGCATCAAAAC	AAAGCGCCTCTTTTCCCTGA
fliA	GGGTGATGGAAGCCATCGAA	TGTGTAACTGACTGACCCGC
fliC	CAAAACCGTCTGGATTCCGC	ATTGGACACTTCGGTCGCAT
motA	GTTTGGTCGCAAAACGCTCT	TCCTCGGTTGTCGTCTGTTG
flhD	AAGATTCCCGCGTTGACGAT	AGCGCTTCTTCAGGCTGATT
flhC	ATGCTGCCATTCTCAACCGA	GCTTTGATCACCGCATCGAC
cyaA	CCAGGTAAAGTGACGCTGGT	CAATATTTGGCGCGCGGTTA
